# Human platelet lysate enables robust expansion of tumor infiltrating lymphocytes from endometrial cancer

**DOI:** 10.3389/fimmu.2026.1873651

**Published:** 2026-10-14

**Authors:** Willemiek Iwema, Nienke van Rooij, Lena Rockstein, Annechien Plat, Refika Yigit, Joost Bart, Bahez Gareb, Hans W. Nijman, Edwin Bremer, Marco de Bruyn

**Affiliations:** 1 Department of Obstetrics and Gynaecology, University Medical Center Groningen, University of Groningen, Groningen, Netherlands; 2 Department of Hematology, University Medical Center Groningen, University of Groningen, Groningen, Netherlands; 3 Department of Pathology, University Medical Center Groningen, University of Groningen, Groningen, Netherlands; 4 Department of Pathology, Leiden University Medical Center, Leiden, Netherlands; 5 Department of Clinical Pharmacy and Pharmacology, University Medical Center Groningen, University of Groningen, Groningen, Netherlands

**Keywords:** endometrial cancer, GMP manufacture, human platelet lysate, human serum, tumor infiltrating lymphocytes

## Abstract

**Background/objectives:**

Tumor infiltrating lymphocytes (TIL) therapy has yielded promising results in melanoma patients, with an approved product available. TIL therapy is of similar interest to a number of additional malignancies, including endometrial cancer (EC). For EC specifically, the tumor is well infiltrated with TILs and has accessible tumors as starting material for TIL isolation/expansion. However, the TIL manufacturing process currently relies on human serum (HS), which is difficult to source and has considerable batch-to-batch variation. Here, we explored the feasibility of using human platelet lysate (HPL) as a good manufacturing practice (GMP)-compatible alternative.

**Methods:**

EC material was obtained following surgery and used to expand TILs in pre–rapid expansion protocols (pre-REP) and REP phases, comparing HPL- and HS-containing media. Tumor tissue from 10 EC patients, representing both high and low tumor mutational burden histotypes was used. Spatial TIL infiltration was assessed using immunohistochemistry. After expansion, TIL phenotype was determined using flow cytometry for CD3, CD56, CD8, CD39, and CD103. TILs from the REP were further characterized with spectral flow cytometry using markers for differentiation, activation, co-stimulatory receptors, and immune checkpoints.

**Results:**

TILs expanded comparably using both HPL- and HS-media during pre-REP and REP in terms of cell yields. All tumors showed an excluded spatial immune phenotype. Immunophenotypes were consistent across media conditions. Spectral cytometry revealed patient-specific clustering unaffected by supplement type.

**Conclusions:**

TILs can be successfully expanded from EC using a fully GMP-compatible HPL protocol. Our findings may serve as a foundation for further improvements of existing GMP-compliant TIL manufacturing protocols.

## 1 Introduction

Tumor infiltrating lymphocytes (TIL) therapy has emerged as a highly promising treatment in oncology, demonstrating encouraging results in solid tumors, such as melanoma, cervical cancer, renal cell carcinoma, and non-small cell lung cancer (1–5). In melanoma, TIL therapy was effective in patients who had progressed after treatment with immune checkpoint inhibitors (ICI) (4). Due to its clinical efficacy in melanoma, the first TIL therapy was approved by the FDA in 2024 for the treatment of unresectable or metastatic melanoma (4).

Endometrial cancer (EC) is the most common gynecological malignancy in high-income countries and is characterized by a rising global incidence and mortality (6). For patients with advanced or recurrent microsatellite instability-high or mismatch repair-deficient EC, current standard treatment involves ICIs. However, as in melanoma, not all patients respond to ICI. Furthermore, in EC the tumor is well infiltrated with TILs and has accessible tumors to source starting material for TIL isolation, and consequently, EC represents a logical indication for TIL therapy (7–10).

A TIL product is classified as an advanced therapy medicinal product (ATMP) and should therefore be manufactured under good manufacturing practice conditions (GMP). The manufacturing of TILs starts with a solid piece of tumor tissue obtained during surgery, and consists of two phases: pre-rapid expansion protocol (pre-REP), and the rapid expansion protocol (REP). The pre-REP phase involves *ex vivo* expansion of T cells through digestion of the tumor tissue and incubation with interleukin-2 (IL-2), with the aim of expanding tumor-reactive T cells. TILs are subsequently expanded to therapeutic cell numbers during the REP phase, in which pooled pre-REP TILs are cultured with anti-CD3 and IL-2, and activated using irradiated peripheral blood mononuclear cells (PBMC) feeder cells to drive expansion. During the manufacturing process, in-process control (IPC) and quality control tests are performed to warrant product quality and safety.

Following a lymphodepleting chemotherapy regimen, the expanded TILs are administered intravenously to the patient, with concurrent IL-2 administrations to promote *in vivo* TIL expansion and enhance the antitumor activity. Current TIL manufacturing processes utilizes human serum (HS), which presents challenges related to limited availability and, more importantly, substantial batch-to-batch variation. For GMP compliance, raw materials with limited batch-to-batch variability are desired, ensuring a standardized quality and manufacturing process.

To address these limitations, we investigated whether human platelet lysate (HPL) could effectively replace HS in TIL expansion protocols. HPL is more readily available, has reduced batch-to-batch variation when using large-scale standardized methods, and has shown comparable activity to HS in e.g. stem cell culture (11–13). Currently, HPL is available in clinical-grade and already used in clinical trials for CAR-T cell manufacturing under GMP conditions (14–16).

We evaluated the feasibility of isolating and expanding TILs from EC tumor samples using HPL as a substitute for HS. The critical quality attributes as well as phenotypic properties were extensively characterized to assess the impact of HPL supplementation during the manufacturing process.

## 2 Materials and methods

### 2.1 Human material

Patients were enrolled in a clinical protocol approved by the Institutional Medical Ethical Board. Informed consent was obtained from all patients before tissue collection. Tumor samples were acquired during a standard-of-care surgical procedure, and routine pathologic assessment confirmed all included tissue as endometrial cancer. Tissue collection occurred between June and September 2023 for TIL cultures. Allogeneic peripheral blood mononuclear cells, called feeder cells, were derived from apheresis of healthy donors at the Sanquin blood bank in the Netherlands. All donors underwent screening for infection markers, including hepatitis B, hepatitis C, hepatitis E, HIV, and syphilis (RPR). Apheresis samples were purified using Ficoll-Paque (Cytiva) density gradients and subsequently cryopreserved.

### 2.2 Pre-rapid expansion protocol

TIL cultures were established according to previously described protocols (2, 3, 17) with modifications. Tumor tissue was aseptically excised during surgery. A portion of the tissue was transferred to a tube containing RPMI medium (Life Technologies) with 10% fetal bovine serum (FBS) (Capricorn Scientific). The tissue was dissected into small fragments in a laminar flow cabinet and transferred into a GentleMACS C-tube (Miltenyi) containing 5–10 ml digestion medium. Digestion medium consisted of TexMACS medium (Miltenyi) supplemented with 12,6 E/ml pulmozyme (Roche). The tissue suspension was dissociated using a GentleMACS Dissociator (Miltenyi). Program h_tumor_01 was run 3–4 times, and the tissue suspension was subsequently filtered through 70 µm cell strainers (Falcon) to remove undigested debris. A Ficoll density gradient was performed to enrich for lymphocytes. After washing with PBS (Apotheek A15), the cell digest was counted, and viability was assessed using a disposable C-Chip Neubauer improved counting chamber (NanoEnTek) with trypan blue (Sigma Aldrich) staining. The cells were plated in a 24-well tissue culture plate at a density of 1x10^6^ total cells per well in 1 mL medium. The culture medium consisted of 90% TexMACS medium, 50 µg/ml gentamicin sulfate (Centrafarm), 6000 U/mL interleukin-2 (IL2) (Novartis), 10% human AB serum (HS; MP Biomedicals) or 10% human platelet lysate (HPL; ELAREM Perform 1 research grade; PL BioScience), and 0.6 U/ml heparin sulfate (Apotheek Albert Schweitzer ziekenhuis, Dordrecht) when using HPL. The plates were incubated in a humidified incubator at 37 °C with 5% CO_2_.

Cultures were maintained for 2–4 weeks, with biweekly inspection under a microscope. Medium was replaced with fresh medium (50% change) once a week. When the well became too full, the culture was resuspended and split at a ratio of 1:1 to 1:2. When sufficient TIL growth was achieved, defined as multiple wells showing high cell density, indicated by the formation of lymphocyte clumps, the cultures were cryopreserved using 90% FBS and 10% DMSO and stored in liquid nitrogen till further use. TIL cultures were excluded for further analysis if there was less than 2 times fold expansion after 4 weeks of culturing.

### 2.3 Rapid expansion protocol

The rapid expansion of pre-REP TIL cultures was performed using a previously described protocol (3). TILs were thawed one day before REP initiation with FBS and cultured in a 24-wells plate, using 10% HS or HPL medium supplemented with 6000 IU/ml IL2. On the day of REP initiation, feeder cells were thawed, resuspended in TexMACS medium supplemented with 50 µg/ml gentamicin sulfate, 6000 U/mL interleukin-2, 10% HS or HPL, and 0.6 U/ml heparin sulfate when using HPL. After irradiation at 40 Gy (IBL 637 Cesium-137y-ray machine), the feeder cells were mixed with TILs at a 1:200 TIL-to-feeder cell ratio, using 0.1x10^6^ TILs. The TIL-feeder mixture was first cultured in T25 flasks, in an upright position for the first 7 days, with 20 ml medium containing 95% TexMACS medium with 5% HS or HPL, 50 µg/ml gentamicin, 3000 IU/ml IL-2 and 30 ng/ml anti-CD3 (OKT3; BD Biosciences). In the case of HPL medium, also 0.6 U/ml heparin sulfate was added. The cultures were incubated at 37 °C in 5% CO_2_ for 14 days. Medium was changed on day 5 or 6, replacing 14 ml with fresh medium without OKT3. From day 5 or 6 onward, cells were checked every 2–3 days and split if necessary. After expansion, all samples were analyzed by flow cytometry and cryopreserved using FBS with 10% DMSO for future use.

### 2.4 Conventional flow cytometry

After cryopreservation, TILs were thawed in FBS, washed with PBS 2% FBS, and incubated for 30 min on ice in the dark with the following antibodies: CD56 PE (eBioscience), CD8 APCeFluor780 (eBioscience), CD3 BV421 (BD Biosciences), CD4 PerCp Cy5.5 (eBioscience), CD39 APC (Thermo Fisher) and CD103 FITC (BD Biosciences). Samples were washed twice with PBS 2% FBS and kept on ice till analysis. Before analysis on the Agilent NovoCyte Quanteon flow cytometer, Propidium Iodide (PI, Sigma) staining was used to discriminate live/dead cells. UltraComp ebeads (Thermo Fisher) were used for compensation controls. The data was analyzed using Cytobank.

### 2.5 Spectral flow cytometry

From TIL samples of different manufacturing conditions, 2x10^6^ cells were collected, washed with and resuspended in 100 µL PBS. The viability marker LIVE/DEAD™ Fixable Blue dye (Thermo Fisher) was added (1 µL) and incubated for 20 min at room temperature (RT) in the dark. Samples were washed once with PBS + 5% FBS and blocked for 10 min at RT with 10 µL TruStain FcX, followed by an extra incubation of 10 min with 5 µL of CellBlox blocking buffer (Thermo Fisher) and TCRγδ antibody. After washing with PBS + 2% FBS, samples were stained for 10 min at 4 °C with 118.5 µl antibody mix (**Supplementary Table 1**) containing 10 µL Brilliant Stain Buffer Plus (USUS Biosciences, San Jose, CA, USA). Following staining with the antibody mix, samples were washed three times with and resuspended in 200 µl PBS + 2% FBS. All washing steps were performed at 300×g for 5 min. An overview of all antibody clones, suppliers, and titrated volumes are indicated in **Supplementary Table 1**. Samples were measured on a 5-laser Aurora spectral flow cytometer (Cytek Biosciences, Fremont, CA, USA) and data were analyzed using OMIQ flow cytometry software (Dotmatics, Boston, MA, USA).

### 2.6 Analysis spectral flow data

Samples were imported into OMIQ and subjected to data cleanup using the FlowAI function with the following settings: Anomaly Detection Method=All Methods (Flow Rate, Signal Acquisition, Dynamic Range), Time Feature=Time, Second fraction FR = 0.1, Alpha FR = 0.01, Changepoint Detection Penalty FS = 500, Max Changepoints FS = 3, Dynamic Range Check Side FM=Both, upper and lower limit, Negative Value Removal Method FM = 1. FlowAI-”passed events” were used for further analysis, and all samples were subsampled accordingly. Dimensionality reduction and visualization were performed in OMIQ on 100000 Live (viability dye negative) subsampled events per file *via* Uniform Manifold Approximation and Projection (UMAP), with the settings as follows: Neighbors=15, Minimum Distance=0.4, Components=2, Metric=Euclidean, Learning Rate=1, Epochs=200, Random Seed=6237. All markers were selected as clustering features aside from the viability dye. Clustered Heatmap was performed in OMIQ on 100000 Live (viability dye negative) subsampled events per file, with the following settings: Measure=Medians, Filter=CD3+CD45+, Cluster Rows, Row Distance=euclidean, Row Linkage=ward, Cluster Columns, Column Distance=euclidean, Column Linkage=ward. All markers were selected as clustering features aside from the viability dye, CD3, and CD45. The exhaustion score of TIL samples and three reference benchmark CD19 CAR-T cell products was determined as described by Weber et al. (18), but using the integrated MFI (iMFI = % positive cells x MFI) with both, percentage of positive expression and MFI data exported from OMIQ for the markers PD-1, TIM-3, LAG3, CD244, CTLA-4, BTLA and CD39.

### 2.7 Immunohistochemical analysis and scoring spatial immune phenotypes

Immunohistochemical (IHC) analysis for CD3 and CD8 was performed on tumor tissue sections derived from the same resection specimens used for TIL expansion. All IHC staining procedures were conducted using the fully-automated Benchmark ULTRA platform (Roche, Ventana Medical Systems, Oro Valley, AZ, USA) under standardized laboratory conditions accredited following the ISO15189 quality system. In short, paraffin tissue sections (3 µm) were incubated with antibodies against CD3 (R.T.U, Ventana, 2GV6), and CD8 (1:20, Dako, C8/144B). As external control, a suitable tissue section was added to each slide. The tumor invasive margins were evaluated by a pathologist according to previously described spatial immune phenotypes, namely immune-desert, immune-excluded, and immune-inflamed (19, 20).

### 2.8 Statistical analysis

Statistical analyses were performed using Graphpad Prism (version 10.2.3 for Windows, GraphPad Software, Boston, Massachusetts USA, www.graphpad.com). Differences in end-cell counts in the pre-REP phase comparing HS and HPL were tested using the Mann–Whitney test. The paired effect estimate for TIL expansion was calculated as the ratio of the mean difference to the difference in standard deviations using (mean HS - mean HPL)/(SD HS - SD HPL). Differences in phenotypes were tested using the Wilcoxon test for paired samples or the paired t-test. All analyses in the REP phase were performed using the Friedman test followed by Dunn’s *post hoc* test. A *p*-value of <0.05 was considered statistically significant.

## 3 Results

### 3.1 Patient characteristics

EC tissue samples were collected from ten patients who underwent standard-of-care hysterectomy between June and September 2023. Detailed clinicopathological characteristics of the patients are presented in **Table 1**. The median age was 67 years (range: 52–79 years). Most tumors were of non-endometrioid histotype (70%). Six out of ten patients had FIGO 2009 stage 1 disease (60%). Tumors with low tumor mutational burden (TMB), e.g. with p53 mutations or classified as the NSMP histotype, were most common (60%).

**Table 1:** Characteristics of the patients who underwent hysterectomy and from whom the EC samples were obtained.

Patient characteristics (n=10)	
Age at enrolment (years)	
Median (range)	67 (52-79)
Histotype (n (%))	
Endometrioid	3 (30)
Non-endometrioid	
Serous adenocarcinoma	3 (30)
Mixed adenocarcinoma^a,b^	2 (20)
Adenosarcoma	1 (10)
Undifferentiated uterine sarcoma	1 (10)
Grade (n (%))	
Endometrioid	
Grade 3	3 (30)
FIGO stage^c^ (n (%))	
I (IA – IC)	6 (60)
II (IIA – IIB)	0 (0)
III (IIIA – IIIC)	4 (40)
Molecular subtype (n (%))	
TMB high	
POLE mutation	1 (10)
MMR deficient^d^	3 (30)
TMB low	
P53 mutant	4 (40)
NSMP^e,f^	2 (20)

^a^ EC674 60% clear cell and 40% serous. ^b^EC687 70% endometrioid and 30% clear cell. ^c^The revised 2009 FIGO staging for endometrial cancer was used for staging patients following standard-of-care hysterectomy. ^d^All 3 patients MLH1 and PMS2 deficient. ^e^EC703 ER negative. ^f^EC693 some small areas of slightly positive ER. FIGO, Fédération Internationale de Gynécologie et d’Obstétrique; TMB, tumor mutational burden; POLE, DNA polymerase epsilon; MMR, mismatch repair; and NSMP, no specific molecular profile.

### 3.2 Expansion and phenotype of HS- and HPL-expanded pre-REP TILs

We observed comparable TIL expansion and phenotypic profile in the pre-REP phase when comparing HPL medium and HS medium (**Figures 1a, b**). We were able to expand TILs from 7 out of 10 HS samples (70%) and 6 out of 10 HPL samples (60%), for both TMB-high and TMB-low tumors, even though TMB-low tumors generally exhibit fewer TILs in the tumor microenvironment (TME) (**Figures 1b–g, i**). There were no significant differences between TILs expanded using HS medium and HPL medium (*p* = 0.84, **Figure 1b**). The mean number of expanded TILs was 43.79 x10^6^ cells (SD: 31.1, range: 13-99x10^6^) in HS medium and 60.03 x10^6^ cells (SD: 48.43, range: 12.8-134x10^6^) in HPL medium, corresponding to a mean-to-standard deviation difference ratio of 0.95. TILs were expanded for a mean of 21 days (range, 15–29 days). There were no significant differences between cell numbers when HPL and HS culture was compared within TMB-high (*p* = 0.5) or TMB-low tumors (*p* = 0.5) (**Figure 1b**).

**Figure 1:**
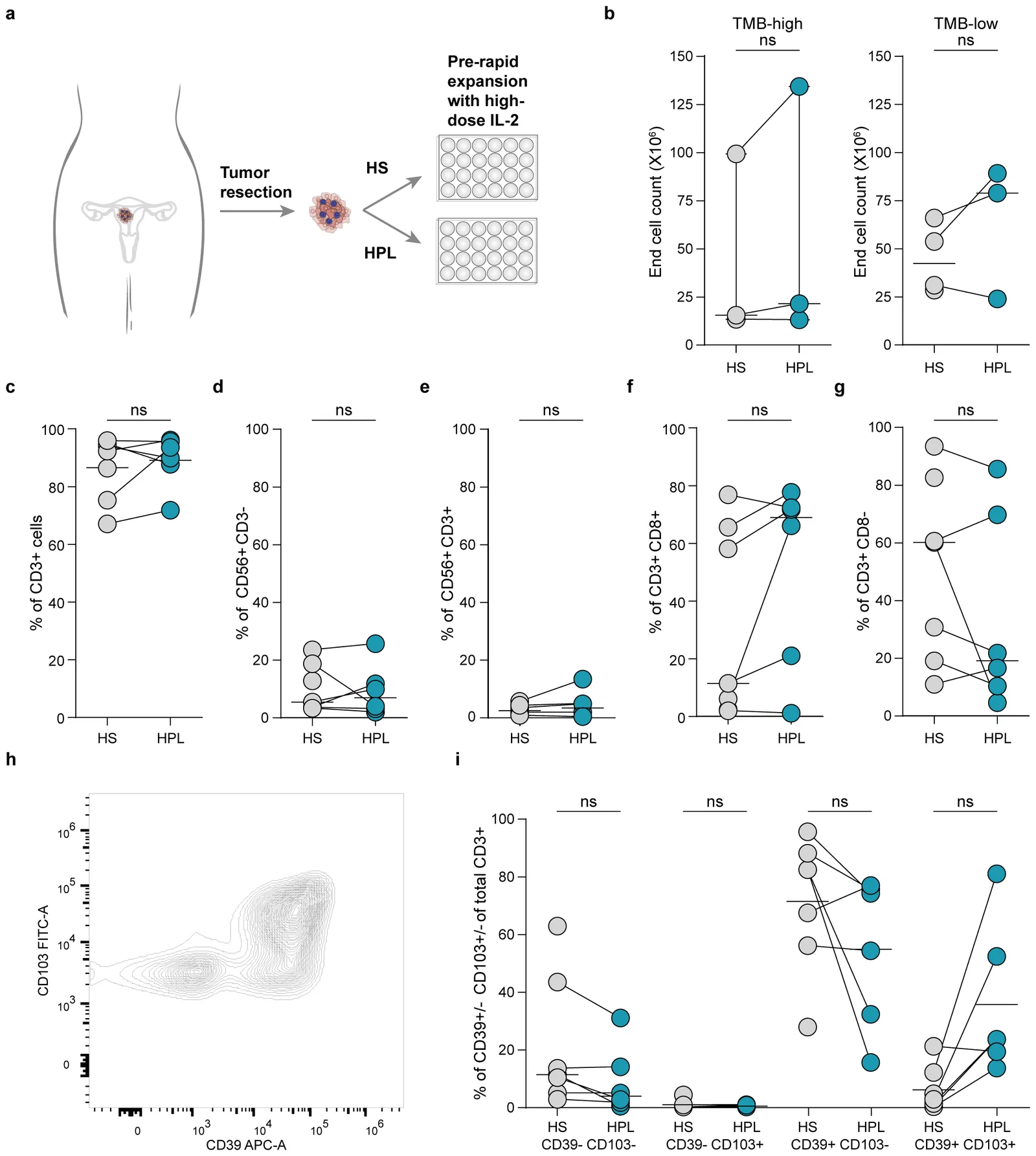
Pre-rapid expansion and phenotype of TILs from endometrial cancer cultured in HS and HPL medium. **(a)** Schematic overview of the experimental setup. **(b)** End cell count of TILs after pre-REP culture in HS medium (grey) or HPL medium (blue) for TMB-high (n=3, left) and TMB-low (n=4, right) histotypes. Data from the same patients are horizontally connected. Statistical significance was assessed using the Mann–Whitney test. **(c–g, i)** Phenotypic analysis of TILs (HS n=7, HPL n=6) after pre-REP including the percentages of CD3+ T cells **(c)**, CD56+CD3- NK cells **(d)**, CD56-CD3+ NK T cells **(e)**, CD3+CD8+ T cells **(f)**, CD3+CD8- T cells **(g)**, and CD39^+^CD103^+^ (tumor-reactive), CD39^−^CD103^+^ (tissue-resident), CD39^+^CD103^−^ (exhausted/activated), and CD39^−^CD103^−^ (non-resident) cells of total CD3+ cells **(i)**, as measured by conventional flow cytometry. Data from the same patients are horizontally connected. Statistical significance was assessed using the Wilcoxon signed-rank test. **(h)** Representative gating strategy for CD39/CD103 flow cytometry analysis. HS human serum, HPL human platelet lysate, IL-2 interleukin-2, TMB tumor mutational burden, ns not statistically significant.

Phenotypic analysis of the expanded TILs was performed using flow cytometry. For both HS and HPL, over 85% of cells were CD3^+^ T lymphocytes with no significant difference in both culture conditions (*p* = 0.84; mean HS 86.58% and mean HPL 89.16%) (**Figure 1c**). CD3^-^CD56^+^ natural killer (NK) cells and CD3^+^CD56^+^ NKT cells did not significantly differ between HS and HPL conditions (NK cells *p* = 0.84, mean HS 10.18%, and mean HPL 9.43%, and NKT cells *p* = 0.43, mean HS 2.84%, and mean HPL 4.38%; **Figures 1d,e**). Furthermore, no significant differences were observed when comparing both medium conditions for CD8+ T cells (CD3^+^CD8^+^), mean HS 31.79%, mean HPL 51.69%, with *p* = 0.16, and CD4^+^ T cells (CD3^+^CD8^-^), mean HS 51.08, mean HPL 34.80, with *p* = 0.44 (**Figures 1f, g**).

Next, the expression of tumor reactivity markers CD39 and CD103 within the total CD3^+^ TIL population was analyzed (21–23). Across all cell subsets, no significant differences were detected between HS and HPL including CD39^−^CD103^−^ (*p* = 0.13, mean HS 21.44%, mean HPL 9.28%), CD39^−^CD103^+^ (*p* = 0.47, mean HS 0.86%, mean HPL 0.42%), CD39^+^CD103^−^ (*p* = 0.10, mean HS 71.50%, mean HPL 54.92%), and CD39^+^CD103^+^ (*p* = 0.06, mean HS 6.19%, mean HPL 35.74%) (**Figures 1h, i**).

### 3.3 Spatial immune phenotype of tumors used for TIL expansion

To investigate whether differences in baseline TIL infiltration might be associated with the ability to generate TIL cultures, we performed CD3 and CD8 immunohistochemical (IHC) staining on tumor slides of all patients, from whom tissue was used to expand TILs. The stained slides were scored and classified according to previously described spatial immune phenotypes: immune-desert, immune-inflamed, and immune-excluded. All tumors (10/10) exhibited an immune-excluded spatial phenotype for both CD3 and CD8 (**Figure 2**), characterized by accumulation within the tumor-associated stroma and along the borders of epithelial islets. In the samples in which TIL expansion was unsuccessful (EC674, EC702, and EC703), CD3- and CD8-positive TILs were present in the tumor tissue at baseline(**Figure 2**).

**Figure 2:**
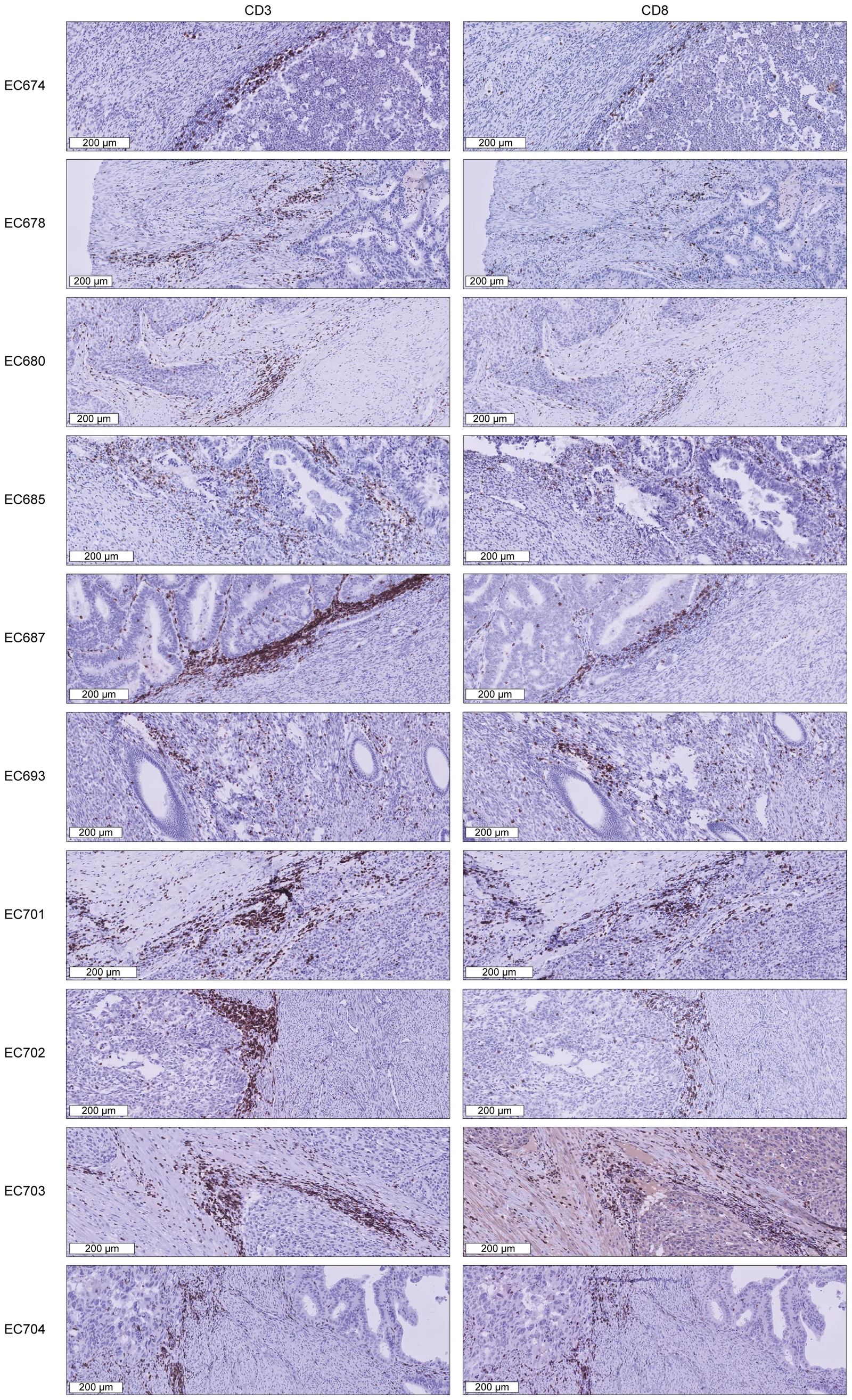
Spatial CD3 and CD8 T cell infiltration in endometrial cancer samples used for TIL cultures. Representative images of the tumor invasive front. Single immunostaining of CD3 (left) and CD8 (right). White rectangles indicate 200 μm. For EC693, only primary tumor material was available for staining, whereas recurrent tumor material was used for TIL expansion.

### 3.4 Rapid expansion protocol and phenotype analysis

We next expanded our analysis of HPL versus HS to include a 2-week REP phase, where we tested 3 TIL samples. TILs that were initially expanded in either HS or HPL medium during the pre-REP phase (**Figure 1**) were cultured in either HS or HPL medium during the REP. This resulted in four conditions: pre-REP HS followed by REP in HS, pre-REP HS followed by REP in HPL, pre-REP HPL followed by REP in HS, and pre-REP HPL followed by REP in HPL (**Figure 3a**). The expanded samples included two TMB-high tumors (EC680, and EC701) and one TMB-low tumor (EC704). Fold expansion was assessed on days 7, 10, 12, and 14 of the REP. In general, HPL and HS cultures were comparable with no significant differences in fold expansion observed across the four medium conditions (*p* = 0.15) (**Figure 3b**). For EC680, pre-REP HS, REP HPL showed a significantly higher fold expansion compared to pre-REP HPL, REP HS (*p* = 0,0197). This difference was consistent across all measured timepoints.

**Figure 3:**
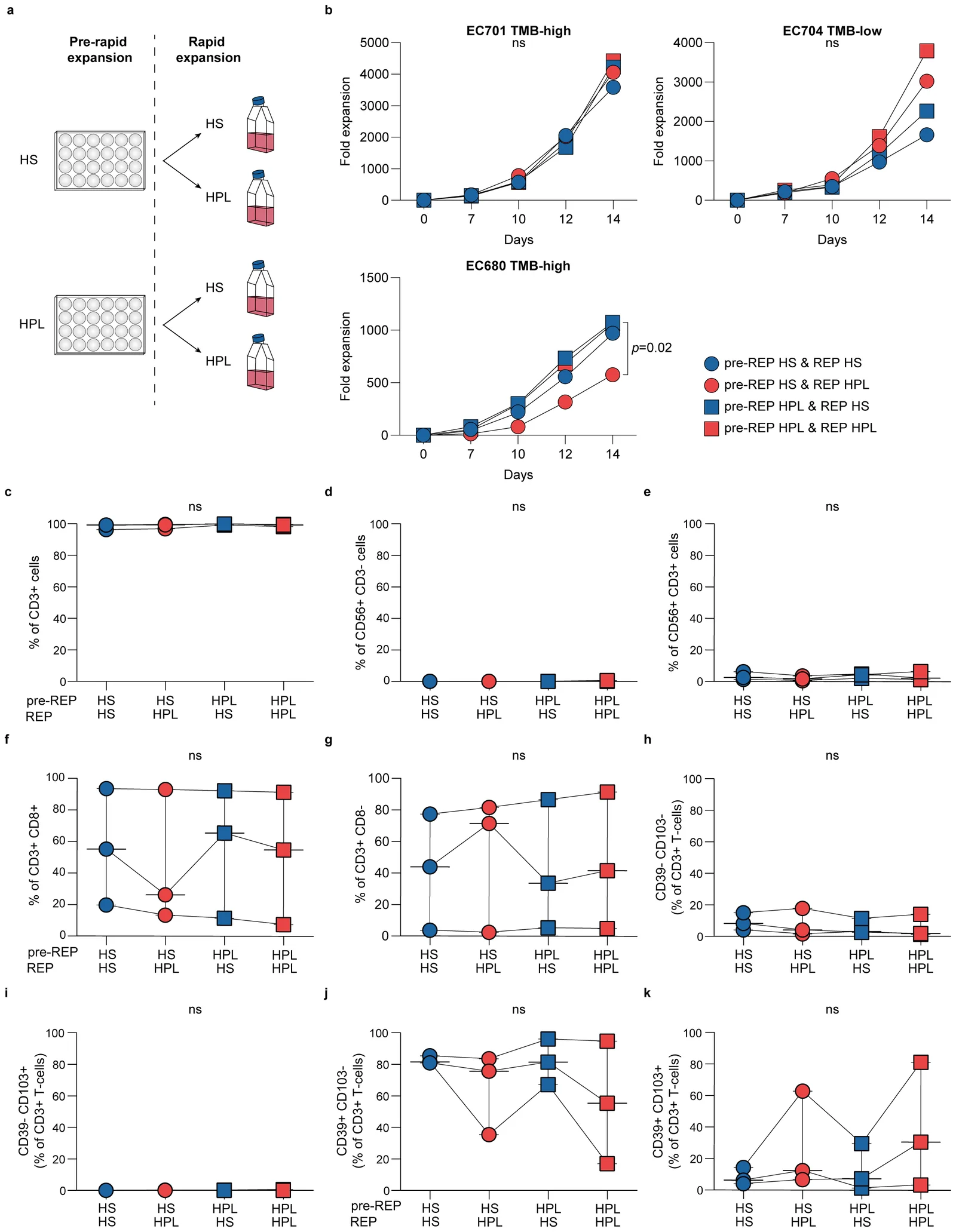
Rapid expansion protocol and phenotype of TILs expanded from pre-REP TILs. **(a)** Schematic overview of the experimental setup. **(b)** Fold expansion of TILs (n=3) cultured in HS or HPL. Pre-REP cultures were grown in HS (circles) or HPL (squares), and REP cultures were expanded in HS (blue) or HPL (red). **(c–k)** Phenotypic analysis of TILs (n=12) after REP, including percentages of **(c)** CD3+ T cells, **(d)** CD56+CD3- NK cells, **(e)** CD56-CD3+ NK T cells, **(f)** CD3+CD8+ T cells, **(g)** CD3+CD8- T cells, and **(h)** CD39-CD103- **(i)** CD39-CD103+, **(j)** CD39+CD103-, and **(k)** CD39+CD103+ cells of total CD3+ T cells, as measured by conventional flow cytometry. Data from the same patients are horizontally connected. Statistical significance was assessed using the Friedman test. Abbreviations: HS human serum, HPL human platelet lysate, TMB tumor mutational burden, ns not statistically significant.

Phenotypic analysis of REP-expanded TILs was performed using the same panel as used for the pre-REP samples. For all four conditions, the population consisted mostly of CD3^+^ T lymphocytes, with no significant differences between the four groups (*p* = 0.053) (**Figure 3c**). For both CD3^-^CD56^+^ NK and CD3^+^CD56^+^ NK T-lymphocytes, no significant difference was observed for the four medium conditions (*p* = 0.45, *p* = 0.34, respectively) (**Figures 3d, e**). Similarly, both CD8^+^ (CD3^+^CD8^+^; *p* = 0.21) and CD4^+^ (CD3^+^CD8^-^; *p* = 0.91) did not differ significantly between groups (**Figures 3f, g**). Finally, we again found no significant differences between the four medium conditions for CD39 and CD103 expression (CD39^−^CD103^−^ (*p* = 0.15), CD39^−^CD103^+^ (*p*>0.99), CD39^+^CD103^−^ (*p* = 0.18), and CD39^+^CD103^+^ (*p* = 0.45) (**Figures 3h–k**).

To gain deeper insight into the cellular composition of post-REP-TILs, we also performed spectral flow cytometry, using a panel of 27 markers used for phenotyping of adoptive T cell therapy(**Supplementary Table 1**). T cells were identified by expression of CD3 and CD45 (**Figure 4a**). In UMAP density scatterplot visualization of spectral flow data, all samples across conditions and patients showed similar localization patterns for CD3^+^ and CD45^+^ cells, indicating largely homogenous phenotypes. Three overarching hierarchical clusters of REP-expanded TILs were clearly separated and defined through expression of CD4, CD8, and γδTCR (**Figure 4b**). The T cell phenotype of all samples, as assessed through hierarchical clustering (**Figure 4c**), was mainly determined by donor-specific clusters, with the closest cluster relations between EC701, EC680, and EC704. Solely EC680 HPL-HS CD3^+^ CD45^+^ cells were more similar to EC701 than other EC680 samples, likely due to the relatively higher expression of T cell maturity markers CCR7 and CD62L, as well as CXCR5 and CD244, and a lower expression of the activation marker CD154 compared to other EC680 samples (cluster in the upper right part of **Figure 4c**). A cluster region defining samples from EC704 had relatively higher expression levels of the potential exhaustion markers TIM-3 and CD39, as well as CD45RO and CD95 (cluster in the lower left part of **Figure 4c**). Overall, across all samples, differences in the CD4:CD8 ratio seem to be determined in a mainly patient-dependent manner (4th and 5th columns of **Figure 4c**). Across all patients and conditions, the range of exhaustion scores of REP-expanded TILs (1.24 – 4.46) is higher than that of independent reference benchmark CAR-T cell infusion products (0.88-1.82) (**Table 2**). However, this seems to be driven by donor-dependency, with EC704 REP-expanded TILs having higher exhaustion scores across all expansion conditions compared to EC701 and EC680. Across the donors, no universal trend for the impact of expansion conditions on the exhaustion score can be found.

**Figure 4:**
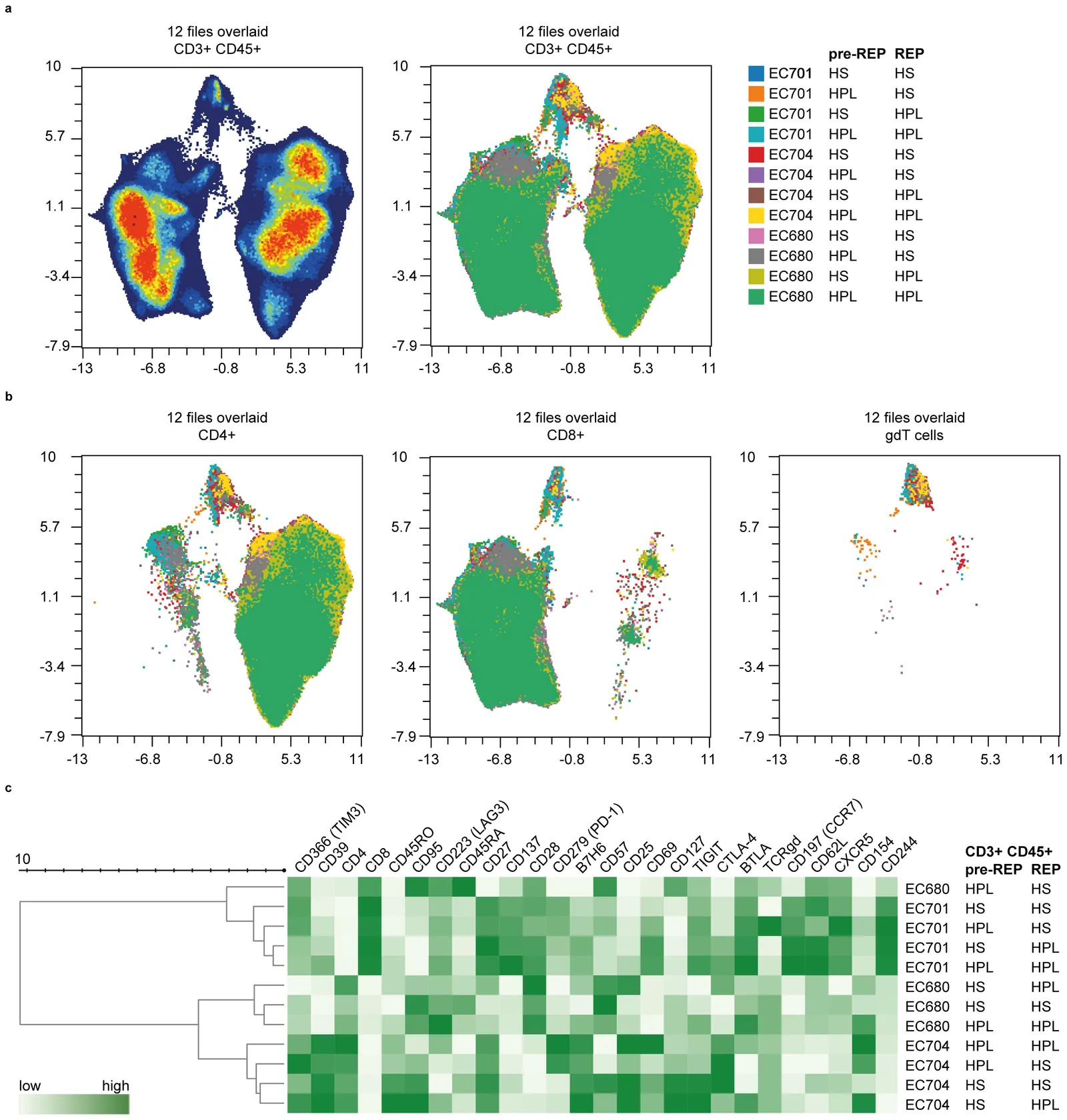
Spectral flow cytometry phenotyping. **(a)** UMAP density plot of TIL samples following different manufacturing regiments and cluster representation per sample within the UMAP. Events displayed are CD3+CD45+. **(b)** UMAP cluster representation per sample filtered for CD4+, CD8+ and TCRγδ+ events, respectively. **(c)** Clustered heatmap filtered for CD3+CD45+ events, with color gradient indicating relative marker expression (white = low, dark green = high).

**Table 2:** Exhaustion scores post-REP TILs compared to CAR-T cells.

Exhaustion scores					
	HS-HS TIL	HPL-HS TIL	HS-HPL TIL	HPL-HPL TIL	CAR-T
EC701	1.35	1.91	2.14	2.99	0.88
EC704	4.02	3.82	4.46	4.31	1.33
EC680	1.35	1.56	1.48	1.24	1.82

## 4 Discussion

The present study demonstrates the feasibility of isolating and expanding TILs from EC samples using HPL as a substitute for HS. We successfully expanded TILs from both TMB-high and TMB-low tumors during both the pre-REP and REP phases. Importantly, expansion in HPL-based medium yielded comparable phenotypic profiles to those obtained with HS. In the pre-REP phase, cultures were predominantly composed of CD3+ T lymphocytes, with no significant differences in the CD4/CD8 ratio between the medium conditions. Post-REP phenotypes clustered mainly based on patient characteristics and were minimally influenced by the culture medium.

HPL was initially developed to support the clinical-grade expansion of mesenchymal stromal cells (MSC) (24). Compared with FBS, multiple studies have shown that HPL offers several advantages for MSC culture. Cultures supplemented with HPL have accelerated proliferation and enhanced cell viability while preserving essential MSC features such as immunophenotype, differentiation capacity, clonogenic potential, and genomic stability (25). HS and HPL differ in their composition, most notably in growth factor composition. HPL contains higher levels of growth factors such as platelet-derived growth factor (PDGF), insulin-like growth factor (IGF), and fibroblast growth factor (FGF), which, among other effects, contribute to enhanced cell proliferation, a positive effect for TIL culture (14, 26). To our knowledge, this is the first study to evaluate the use of HPL-based culture medium for GMP-compatible TIL expansion protocols in EC.

In CAR-T cell research, HPL has also shown several advantages over HS and FBS. CAR-T cells cultured in HPL-containing medium maintain higher proportions of naïve and central memory T cells, which are associated with improved persistence and anti-tumor activity *in vivo* (14, 15). Similarly, HPL-supplemented cultures of CAR-Vδ2 T cells demonstrated enhanced expansion, comparable *in vitro* cytotoxicity, and superior *in vivo* persistence compared with FBS-expanded cells (16). In contrast to these findings, our study used T cells expanded from tumor tissue instead of PBMCs, which is the case for CAR-T cell manufacturing. We observed comparable TIL expansion and phenotypic profiles between HPL- and HS-based cultures. This suggests that, while HPL supports efficient TIL growth, its effect on phenotype may be less pronounced than that reported for CAR-T cells. It is essential to note that a relatively small sample size limited our study, and we did not include functional assays to assess tumor reactivity, which should be addressed in future investigations.

Previous studies have reported that HPL-expanded T cells, expanded from PBMCs, show increased proportions of CCR7+/CD45RO+ central memory subsets (15), which are associated with enhanced persistence and antitumor activity. In our study, however, we did not observe culture medium dependent differences in CD45RO and CD197 (CCR7) expression. Instead, these markers varied primarily between individual patients. Similarly, other markers commonly used to define memory subsets, such as CD62L, CD27, and CD127, were also comparable across medium conditions. We also observed comparable expression of exhaustion markers between HPL- and HS-expanded TILs, both as an integrated score, and when analyzed as individual markers. Nevertheless, overall exhaustion scores were higher than benchmark CAR-T products, suggesting the REP expansion does not fully reprogram TILs towards a PBMC-like CAR-T phenotype, and highlighting a potential direction for product improvement. These results suggest that in our cohort HPL does not markedly alter the TIL phenotype.

Our findings confirm that TIL generation from EC tissue is feasible across both TMB-high and TMB-low tumors. This is particularly relevant given the heterogeneity of EC, where TIL infiltration varies widely across molecular subtypes. A previous study reported TIL expansion from both TMB-high and TMB-low EC samples, from mostly endometrioid histotype and grade II tumors, with a success rate of 91% TIL growth, and demonstrated their antitumor activity *in vitro* (27). Our data extend these findings by showing comparable TIL expansion in a more diverse patient population in terms of histological subtypes. Additionally, we demonstrate that similar expansion can be achieved using HPL as a serum substitute, supporting its application in GMP-compatible TIL manufacturing.

A limitation of this study was that no functional testing of HPL-expanded TILs was performed to evaluate tumor reactivity, cytokine production, and cytotoxic potential. It will also be important to assess whether HPL influences the repertoire or persistence of tumor-reactive T-cell clones during expansion. Large-scale validation and comparison across EC subtypes could provide more insight into the robustness and reproducibility of HPL-supported TIL production.

In summary, we demonstrated that TILs can be effectively expanded from EC using HPL-based culture medium, achieving comparable yields and phenotypic profiles to HS-based culture. Ultimately, integrating HPL into TIL manufacturing protocols could facilitate broader translational and support the development of more reproducible and accessible adoptive T-cell therapies.

## Data Availability

The original contributions presented in the study are included in the article/**Supplementary Material**, further inquiries can be directed to the corresponding author/s.
