# Supplementary material for: Human platelet lysate enables robust expansion of tumor infiltrating lymphocytes from endometrial cancer
Source: Front Immunol. 2026 Oct 14;17:1873651. doi: 10.3389/fimmu.2026.1873651 (PMC13713836; doi:10.3389/fimmu.2026.1873651)
Supplement: Supplementary file 1 [file Table1.pdf]

**Supplementary Table 1.** Spectral flow cytometry T cell phenotyping panel

| Marker | Clone | Fluorochrome | Cat. number | Supplier | Titrated volume (µL) |
|---|---|---|---|---|---|
| **TCRγδ** | B1.1 | PerCP-eFluor710 | 46-9959-42 | Thermo Fisher | 5 |
| **CXCR5 (CD185)** | RF8B2 | BV750 | 747111 | BD | 1.25 |
| **CCR7 (CD197)** | G043H7 | BV421 | 353208 | Biolegend | 10 |
| **CD45RA** | 5H9 | BUV395 | 740315 | BD | 1.25 |
| **CD4** | SK3 | NovaFluor Yellow 570 | H001T03Y01 | Thermo Fisher | 2 |
| **CD45** | 2D1 | PerCP | 368506 | Biolegend | 2.5 |
| **LAG3 (CD223)** | 3DS223H | BUV563 | 365-2239-42 | Thermo Fisher | 2.5 |
| **CD62L** | DREG-56 | PerCP-Cy5.5 | 304823 | Biolegend | 10 |
| **CD127** | HIL-7R-M21 | R718 | 566967 | BD | 5 |
| **CD137** | 4B4-1 | PE-Cy7 | 309818 | Biolegend | 5 |
| **CD154** | TRAP-1 | BV480 | 746337 | BD | 5 |
| **CD25** | M-A251 | PE/Fire 700 | 356146 | Biolegend | 5 |
| **CD28** | CD28.2 | BV605 | 562976 | BD | 5 |
| **CD3** | SK7 | BV510 | 344828 | Biolegend | 5 |
| **CD39** | TU66 | BUV661 | 749967 | BD | 5 |
| **CD45RO** | UCHL1 | PE/Dazzle™ 594 | 304248 | Biolegend | 5 |
| **CD69** | FN50 | BV650 | 563835 | BD | 5 |
| **CD8** | SK1 | BUV805 | 612889 | BD | 5 |
| **PD-1 (CD279)** | EH12.2H7 | BV785 | 329930 | Biolegend | 5 |
| **TIM3 (CD366)** | F38-2E2 | PE-Cy5 | 345052 | Biolegend | 5 |
| **CD27** | O323 | APC/Fire 750 | 302846 | Biolegend | 10 |
| **CD95** | DX2 | APC | 305612 | Biolegend | 10 |
| **TIGIT** | 741182 | BUV615 | 752314 | BD | 2 |
| **CD244** | C1.7 | Alexa Fluor 647 | 329510 | Biolegend | 2 |
| **CTLA-4** | BNI3 | BB515 | 566917 | BD | 5 |
| **B7H6** | JAM1EW | Super Bright 436 | 62-6526-42 | Invitrogen | 5 |
| **BTLA** | J168-540 | BUV496 | 750251 | BD | 5 |
